# Hyperprolactinaemia: audit of practice and new guidance

**DOI:** 10.1192/bjo.2021.529

**Published:** 2021-06-18

**Authors:** Benjamin Janaway, Lubna Anwar

**Affiliations:** 1 Barnet, Enfield and Haringey Mental Health Trust; 2 Enfield South Locality Team (Lucas House)

## Abstract

**Aims:**

Hyperprolactinaemia is a problem secondary to antipsychotic use. Current management guidelines are heterogeneous and impractical. We aimed to assess coherence to common themes monitoring and intervention, reasons for failure, and to design new guidance for both general use Barnet, Enfield and Haringey Mental Health Trust (BEHMHT) and beyond.

We hypothesised that performance would be poor and new guidance warranted.

**Background:**

Hyperprolactinaemia is defined as blood prolactin of >530 miu/L in females and >424 miu/L in males, with 49.9% is due to medication. Several agents are deemed higher risk Symptom profiles and risk are idiosyncratic and there are adverse long-term outcomes. Treatment is based on symptom profile and severity and cause. Current guidance is trust specific or advised through The Maudlsey Prescribing Guidelines.

Comprehensive and practical guidance reflecting front-line limitations is lacking. There is no clear delineation of a risk stratified pathway.

**Method:**

We wished to ascertain data on surveillance, aetiology and signpost opportunities for service improvement. We also designed ‘risk strata’ to guide intervention.

A random sample (n30) was selected from Enfield South Locality Team and data captured using local records. No ethical considerations were raised.

A number of audit standards (95%) were developed based on previous guidance and agreed within the team and included frequency of monitoring, time to review and need for further referral.

New guidance was developed based on results, MDT agreement and consultation with medical specialities.

**Result:**

Data (n 30) showed predominant male bias to sample (66%) and average age of 48.87 yrs. Predominant diagnoses were Paranoid Schizophrenia (53.33%) and Schizoaffective disorder (33.33%.) Only 7/30 (23.33%) had undergone testing within the last year.

Of those sampled, 2 (6.667%) had a new diagnoses of Hyperprolactinaemia, one on routine monitoring, one incidentally on admission to hospital. Both were on high risk agents. Both were reviewed and treated within one month. No audit standards were met, but no further referrals were required.

Reasons for failure varied, but included loss to follow-up, no test requested or appointments missed.

**Conclusion:**

Based on these data it was noted that monitoring was poor and reasons for failure varied. New Guidance was developed in response. The scope and validity of this guidance was agreed by MDT and awaits formal ratification.

Re-audit will occur in 2020, and if successful the guidance submitted to other Trusts and RCPSYCH for national use.

No financial interests to declare.

